# Dr. Jerome Cochran

**Published:** 1896-09

**Authors:** 


### Obituary.

---

Dr. Jerome Cochran, of Montgomery, Ala., died at his home in that city, August 18, 1896, of chronic nephritis, aged sixty-four years. Dr. Cochran had been physically enfeebled for some years and was a sufferer from partial hemiplegia, but of late this condition had somewhat improved and in May of this year he was in attendance upon the meeting of the American Medical Association at Atlanta, looking better than for some time previously. It was there remarked by his many friends that his appearance gave promise of a lengthened lease of life.

Dr. Cochran graduated in medicine at the University of Nashville in 1861, and soon afterward entered the confederate army, serving as surgeon until the close of the war. In June, 1865, he established himself at Mobile, where for ten years he occupied teaching chairs in the medical college of Alabama. For the past fifteen years he has resided at Montgomery, where his most conspicuous work was done in relation to public health and state medicine. He drafted the laws pertaining to public medicine in Alabama, was state commissioner of health and chairman of the Alabama state board of medical examiners.

Dr. Cochran was an acknowledged authority on the subject of yellow fever, having, in company with Surgeon-General Woodworth, of the Marine Hospital service, and Dr. S. M. Bemiss, of New Orleans, investigated the yellow fever epidemic of 1878, on which a valuable report was made, remarkable for its painstaking nature and of which Dr. Cochran is believed to be the author. He was a member of the American Public Health Association and expected to attend the meeting at Buffalo during this month. At Atlanta, last May, he was appointed to deliver the address on state medicine before the American Medical Association at Philadelphia next year, and his name was also mentioned prominently in connection with the presidency of the association.

Dr. Cochran was a man of versatile talents, a student of many subjects outside of his own profession, a charming conversationist and a most delightful companion, whose friends and acquaintances, scattered all over the continent, will deplore his death.

